# A crucial role for spatial distribution in bacterial quorum sensing

**DOI:** 10.1038/srep34695

**Published:** 2016-10-04

**Authors:** Meng Gao, Huizhen Zheng, Ying Ren, Ruyun Lou, Fan Wu, Weiting Yu, Xiudong Liu, Xiaojun Ma

**Affiliations:** 1 Dalian Institute of Chemical Physics, Chinese Academy of Sciences, Dalian 116023, P.R. China; 2 University of the Chinese Academy of Sciences, Beijing 100049, P.R. China; 3 College of Environment and Chemical Engineering, Dalian University, Dalian Economic Technological Development Zone, Dalian 116622, P.R. China

## Abstract

Quorum sensing (QS) is a process that enables bacteria to communicate using secreted signaling molecules, and then makes a population of bacteria to regulate gene expression collectively and control behavior on a community-wide scale. Theoretical studies of efficiency sensing have suggested that both mass-transfer performance in the local environment and the spatial distribution of cells are key factors affecting QS. Here, an experimental model based on hydrogel microcapsules with a three-dimensional structure was established to investigate the influence of the spatial distribution of cells on bacterial QS. *Vibrio harveyi* cells formed different spatial distributions in the microcapsules, i.e., they formed cell aggregates with different structures and sizes. The cell aggregates displayed stronger QS than did unaggregated cells even when equal numbers of cells were present. Large aggregates (LA) of cells, with a size of approximately 25 μm, restricted many more autoinducers (AIs) than did small aggregates (SA), with a size of approximately 10 μm, thus demonstrating that aggregate size significantly affects QS. These findings provide a powerful demonstration of the fact that the spatial distribution of cells plays a crucial role in bacterial QS.

Microbial infections can have serious consequences for human health; examples are chronic wounds, otitis media, and periodontitis^1^. The formation of antibiotic tolerant sessile biofilms and the synthesis and secretion of toxic factors, which are regulated by bacterial cell-cell communication (called quorum sensing, QS), play important roles in infections. As a result, research on bacterial QS has increased over the past few decades^2^,^3^. QS is generally considered to be a cell density-dependent phenomenon^4^: that is, microorganisms sense the surrounding cell density, judge whether the cell density is sufficient for a coordinated response, and subsequently alter downstream gene expression. However, research has increasingly indicated that bacteria, as prokaryotes, cannot distinguish cell density from other complex environmental factors^5^. In fact, mass-transfer properties and cell spatial distribution are also important factors that should be considered when studying QS.

Microorganisms exist as social communities in nature, such as biofilms on interfaces or flocs in liquid culture. Microorganisms form cell aggregates by transforming from single cells into a three-dimensional (3D) bacterial community. This aggregate structure influences cell-cell communication by affecting both the mass transfer properties of signaling molecules and the spatial distribution of cells. The mass transfer of signaling molecules specifically determines the ability of microorganisms to sense the local concentration of autoinducers (AIs) and significantly influences bacterial QS. This theory has been demonstrated in both unicellular and multicellular systems^6^,^7^,^8^,^9^,^10^. The spatial distribution of cells, that is, the various cell aggregate structures generated from a fixed number of cells in a finite space, is also thought to influence bacterial cell-cell communication. Both mathematical models^11^ and experimental data support these assumptions. For instance, Connell *et al*. prepared microtraps with 3D-printing technology and regulated the number of cells (*Pseudomonas aeruginosa*) to be as few as 500 in each microtrap. The authors then monitored pyocyanin, a secondary metabolite of *Pseudomonas aeruginosa* that is under strict QS control, by real-time scanning electrochemical microscopy and demonstrated the impact of spatial organization and aggregate size on microbial behavior^12^. However, this method could only aggregate cells together in the microtrap and could not induce the formation of a 3D structure or the generation of different spatial distributions in a finite space.

In the present study, we developed a method to form different cell aggregate structures in a finite space, that is, random distribution and clustered distribution, based on alginate/ε-poly-L-lysine microcapsules, and then characterized the influence of cell spatial distribution on QS. When similar numbers of cells (*Vibrio harveyi*) formed different aggregate structures in a finite space, they displayed different QS responses. In contrast to random distributed cells (no aggregates), clustered distributed cells (cell aggregates) exhibited a stronger QS phenomenon mainly because the cell aggregates restricted the diffusion of AIs and reduced the cell-cell communication distance, which is beneficial for bacterial QS. Moreover, large aggregates (LA) of approximately 25 μm exhibited much stronger QS responses than did small aggregates (SA) of approximately 10 μm, showing that the size of cell aggregates, as regulated by the established 3D model, had a significant effect on QS.

## Results

### The effect of spatial distribution on bacterial quorum sensing

*Vibrio harveyi,* one of the best-studied model organisms in QS studies, has been employed in this study owing to its well-studied QS pathways^13^ and its easily detected bioluminescent QS phenotype. *V. harveyi* produces and responds to three different classes of AIs. Two of which are canonical QS systems: the species-specific HAI-1 (N-(3-hydroxybutyryl)-homoserine lactone), which belongs to the N-acyl homoserine lactone (AHL) family and is commonly used by Gram negative bacteria, and AI-2 (furanosyl borate diester), which is used in inter-species communication^14^. These two AIs are synthesized by the LuxM and LuxS proteins and are recognized by the two membrane-bound hybrid sensor kinases encoded by *luxN* and *luxQ*. The LuxR protein, which is regulated by phosphorylation of the LuxU and LuxO proteins (encoded by the *lux* operon) activates genes required for bioluminescence, biofilm formation and proteolysis and represses genes involved in type III secretion and siderophore production^15^,^16^,^17^.

**Figure 1:**
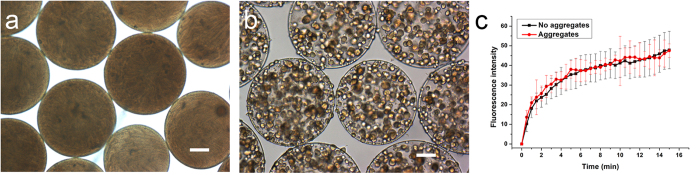
The morphology and mass transfer properties of microcapsules with different spatial distributions of cells. (**a,b**) Optical images of uniformly distributed cells and cell aggregates in microcapsules (bar = 100 μm). (**c**) The mass transfer properties of microcapsules with different spatial distributions of cells. FITC was chosen as a model molecule because it is similar in size to the majority of autoinducers. FITC freely diffused in both types of microcapsules and achieved equilibrium in less than 15 minutes. Error bars represent the standard deviation of five replicates.

The spatial distribution of cells has been suggested to play a crucial role in bacterial QS^5^. In the present study, an experimental model was developed using alginate/ε-poly-L-lysine microcapsules to provide a finite 3D space to entrap a fixed number of cells while enabling the formation of different spatial distributions of cells. An alginate/poly-lysine microcapsule comprises two main components: a core material of calcium alginate gel-sol enclosed by a polyanion-polycation complex membrane as an outer coating of microcapsule. Alginate, which is rich in carboxyl group, can react with the amino group of poly-lysine so as to form a polyanion-polycation complex membrane. The primary function of the poly-lysine is to form a strong complex membrane which should stabilize and strengthen the ionic gel network and reduce the leakage of cells^18^,^19^. The cells in the microcapsules were either uniformly distributed without an aggregate structure (Fig. 1a) or clustered together to form cell aggregates (Fig. 1b). However, the changes in cell structure did not influence the mass-transfer properties of microcapsules. Thus, fluorescein isothiocyanate (FITC, 398 Da), as small as AIs (AI-1 187 Da and AI-2 193 Da), can penetrate inside the microcapsules with similar diffusion rate in both groups and reach equilibrium in a short time as shown in Fig. 1c. That means the cell distribution does not affect the integral mass transfer property of the cell-entrapped microcapsules. Therefore, an experimental model was successfully established using microcapsules with 3D inner structure and similar mass transfer property of membrane for signaling molecules, which can regulate similar cell number into different cell spatial distributions for QS study.

**Figure 2:**
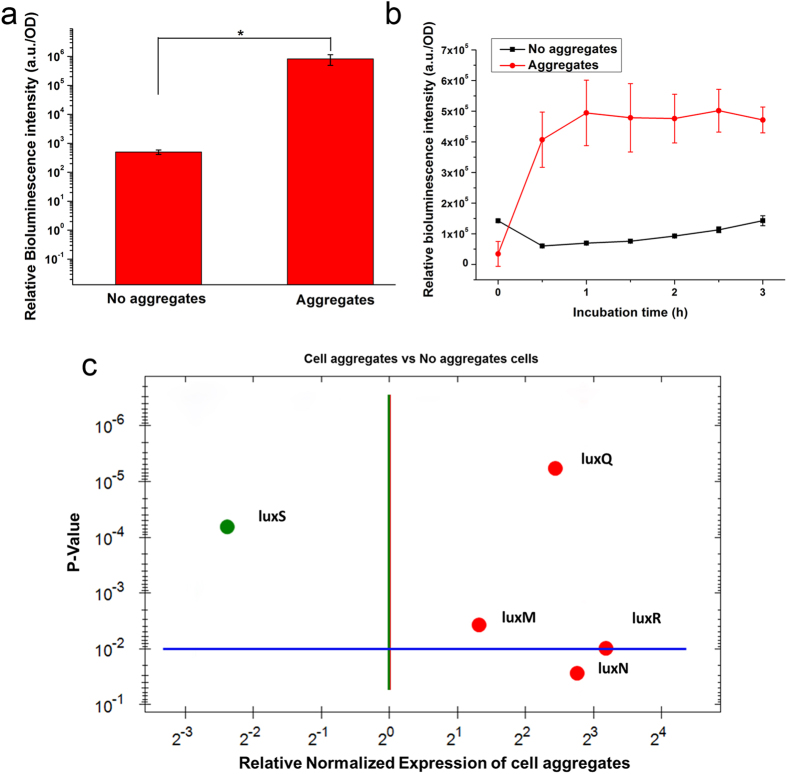
The QS capacities of cells with different spatial distributions in microcapsules. (**a**) The relative bioluminescence intensity (RBI) of *V. harveyi* cells in structures with different spatial distributions. (**b**) The QS response of *V. harveyi* cells in structures with different spatial distributions when an equal volume of sterile supernatant was added as an exogenous AI. (**c**) The expression levels of QS-related genes in different spatial distributions. The RNA polymerase A subunit gene (*rpoA*) was used as a housekeeping gene, and the results were normalized to the no-aggregate group. Genes in green color indicated down-regulated expression and genes in red color indicated up-regulated expression. Error bars represent the standard deviation of three replicates. Significant differences between groups were analyzed by t-test. Single asterisk indicates significant difference (p < 0.05).

The *V. harveyi* cells that formed cell aggregates in the microcapsules exhibited a much higher bioluminescence intensity than did the unaggregated cells (Fig. 2a). Moreover, when the same concentration of exogenous signaling molecules (sterile supernatant from a 12-hour culture of *V. harveyi*) were added for 3 h, the cell aggregates displayed a stronger bioluminescence response than did cells that did not form aggregates (Fig. 2b). Similar results were obtained by real-time quantitative PCR (Fig. 2c). Most of the genes related to *V. harveyi* QS were highly expressed, particularly *luxM/luxN* genes (regulating the AI-1 QS pathway) and *luxR* gene (activating luciferase secretion) (p < 0.05). These results indicate that when the spatial distribution of cells changes, a similar number of cells in the same finite space can display different QS capacities: that is, cell aggregates facilitate bacterial QS.

### The effects of the size of cell aggregates on bacterial quorum sensing

**Figure 3:**
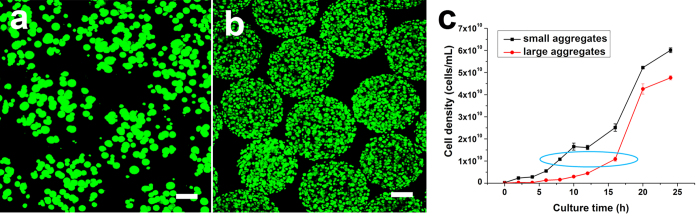
The morphology of cell aggregates with different sizes. (**a**) LA inside the microcapsules, with a size of 27 ± 6 μm. (**b**) SA inside the microcapsules, with a size of 10 ± 2 μm. All fluorescence images were obtained by CLSM, and the cells were stained with a LIVE/DEAD kit (bar = 100 μm). Aggregate size was quantified with ImageJ software. (**c**) The growth curves of microcapsules with large and small aggregates. The time point of which the cell density was similar and cell cycle was both in logarithmic phase was chosen as the experimental subjects (LA and SA groups) for comparison, which was indicated by the blue cycle. That is, cells in LA group reached 1.09 × 10^10^ CFU/mL when cultured for 16 hours and in SA group cells reached 1.08 × 10^10^ CFU/mL when cultured for 8 hours. Error bars represent the standard deviation of three replicates.

**Figure 4:**
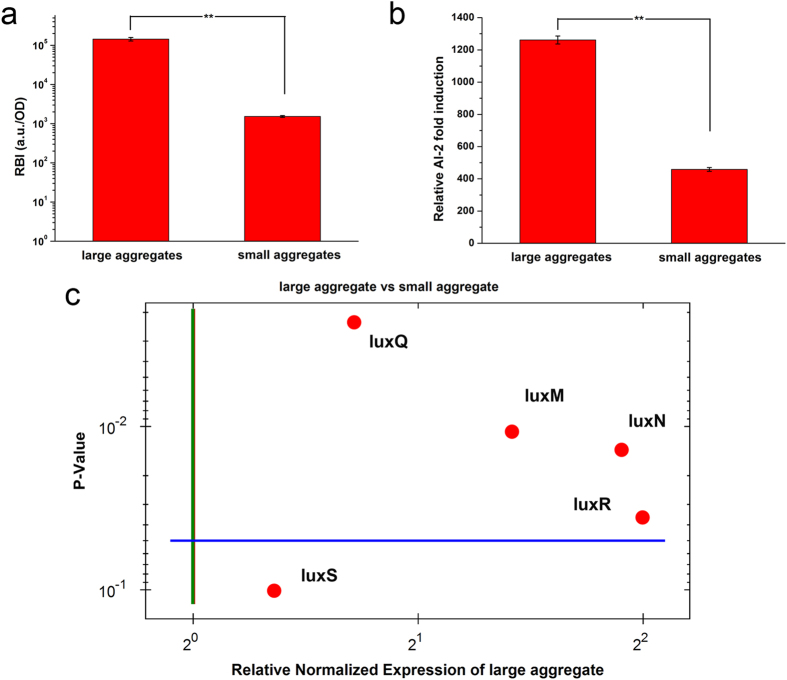
The QS capacities of cells with different aggregate sizes in microcapsules. (**a**) The relative bioluminescence intensity (RBI) of *V. harveyi* cells in aggregate with different sizes. (**b**) The relative AI-2 induction of different aggregate sizes. AI-2 induction was quantified using a biological method employing *V. harveyi* BB170 as a reporter strain, and the supernatant from BB152 was used as a positive control. (**c**) The expression levels of QS-related genes in aggregates with different sizes. The RNA polymerase A subunit gene (*rpoA*) was used as a housekeeping gene, and the results were normalized to the SA group. Genes in green color indicated down-regulated expression and genes in red color indicated up-regulated expression. Error bars represent the standard deviation of three replicates. Significant differences between groups were analyzed by t-test. Single asterisk indicates significant difference (p < 0.05) and double asterisk indicates significant difference (p < 0.01).

Based on the above findings that cell aggregates can significantly improve bacterial QS, further experiments were designed to explore whether the number of cell aggregates or the number of cells in each aggregate had a greater effect on bacterial QS. As shown in Fig. 3, different cell aggregates were prepared by encapsulating 5 × 10^6^ CFU/mL and 5 × 10^8^ CFU/mL *V. harveyi* cells in alginate/ε-poly-L-lysine microcapsules followed by culture for 16 h and 8 h respectively, as at this time they reached the same cell number (Fig. 3c). QS phenomena were then investigated. Because the finite space contained a similar total number of cells and the membranes of the cell-entrapped microcapsules had similar mass-transfer properties (shown in Fig. S1), the LA group (size of aggregates, 27 ± 6 μm) had a smaller number of aggregates but more cells in each aggregate. By contrast, the SA group (size of aggregates, 10 ± 2 μm) had more cell aggregates in the microcapsules but fewer cells in each aggregate. The LA group exhibited higher bioluminescence intensity (Fig. 4a) and secreted more AI-2 (AI-2, Fig. 4b). Genes related to the AI-1 QS pathway (*luxM/luxN*, p < 0.05) or the AI-2 QS pathway (*luxS/luxQ*) were significantly higher expressed in the LA group than in the SA group (Fig. 4c). Taken together, these results demonstrate that the LA group had a much stronger QS phenotype than the SA group.

### The distribution of signaling molecules related to QS

**Figure 5:**
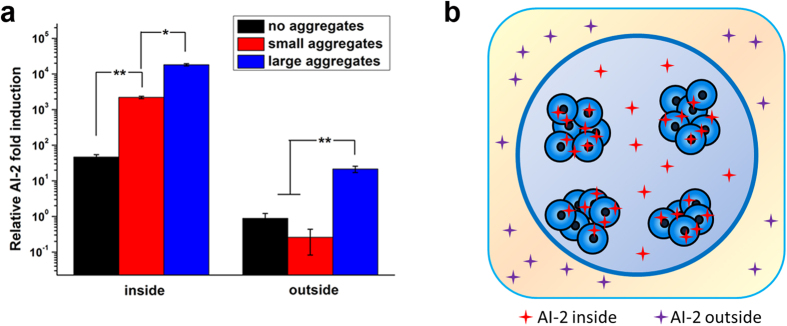
The AI-2 distribution inside and outside microcapsules. (**a**) The concentrations of AI-2 induced in the context of three types of spatial distributions of cells were quantified with a biological method. (**b**) A schematic of the AI-2 distribution. Most of the AI-2 was restricted in the cell aggregates, and the remainder diffused outside the microcapsules. Error bars represent the standard deviation of three replicates. Significant differences between groups were analyzed by t-test. Single asterisk indicates significant difference (p < 0.05) and double asterisk indicates significant difference (p < 0.01).

**Figure 6:**
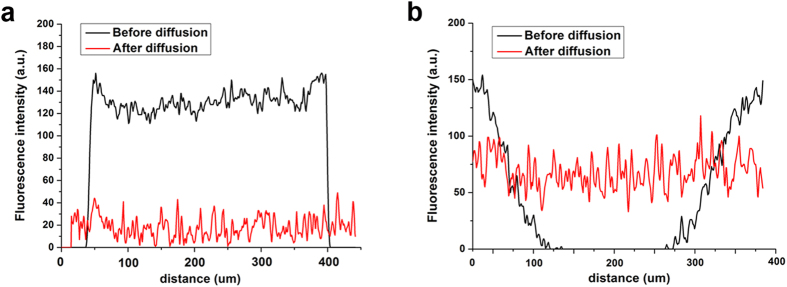
Mass transfer properties of empty microcapsules. (**a**) The fluorescence intensity of FITC before and after outward diffusion. Empty microcapsules were incubated in a FITC solution (0.1 mg/mL) for 4 hours to achieve equilibrium of the fluorescence, and 100 μL of microcapsules containing FITC was then added to 900 μL of saline solution. The FITC inside the capsules immediately diffused outward and reached equilibrium. (**b**) The fluorescence intensity of FITC before and after inward diffusion. A 20-μL aliquot of empty microcapsules was added to 200 μL of FITC solution (10 μg/mL) and immediately photographed by CLSM. The FITC diffused inside the microcapsules and rapidly reached equilibrium. The experiments have been repeated for three times.

**Figure 7:**
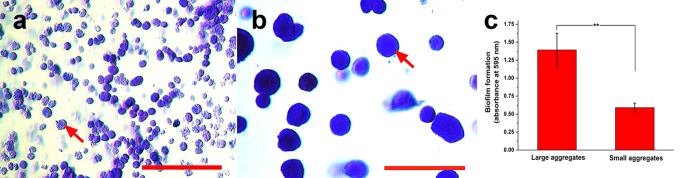
Biofilm formation of the cells with different aggregate sizes in the microcapsules. (**a,b**) The morphology of biofilm generated outside of small and large aggregates, respectively. The biofilm was characterized by the crystal violet staining. Bar = 100 μm. The red arrows indicated the biofilm formed outside of the aggregates. (**c**) Quantification of biofilm in different aggregates group and quantified at the absorbance of 595 nm. Error bars represent the standard deviation of three replicates. Significant differences between groups were analyzed by t-test. Single asterisk indicates significant difference (p < 0.05).

To further elucidate the mechanisms underlying the stronger QS phenotype caused by cell aggregates in the microcapsules, the distribution of signaling molecules related to QS in the microcapsules was examined. The distribution of AI-2 signaling molecules inside or outside the membrane of microcapsules was detected (Fig. 5). The concentration of AI-2s inside was much higher than that outside in all three groups (no aggregates, SA, and LA groups), because a decreased concentration gradient of signaling molecules existed through microcapsules as they were produced by cells. Moreover, the AI-2 concentration inside the microcapsules significantly increased with aggregate formation and size enlargement (Fig. 5). The LA group, with more cells in each aggregate, produced 10 times more AIs both inside and outside the microcapsules than the SA group did, which suggested the spatial distribution of cells in LA group facilitated the secretion of signaling molecules. In general, the substance permeability of microcapsules (mass transfer properties) significantly influences the growth, metabolism and QS of microbial cells. Therefore, FITC, a molecule as small as AIs, could freely diffuse in and out of the empty microcapsules (Fig. 6a,b), suggesting that the membrane of the microcapsules did not restrict the outward diffusion of AIs secreted by the bacteria. As described above, signaling molecules (AI-2) could freely diffuse out of the microcapsules under the concentration gradient when they were secreted and separated from cells. The AI-2 concentration inside of microcapsules was detected after breaking microcapsules and disintegrating the aggregates. As a result, the AI-2 concentration inside of microcapsules with cell aggregates was significantly higher than that without aggregates (Fig. 5a). This difference might be ascribed to the formation of biofilm on the surface of aggregated cells. Obvious biofilm-like structure can be seen outside of both small and large aggregates using crystal violet stain (Fig. 7a,b), and further quantification showed that significant bigger amount of biofilm was formed on the large aggregates (Fig. 7c). In the biofilm, the aggregate became a collective set of cells, and the biofilm structure may have affected the diffusivity of AIs as well.

## Discussion

Extensive efforts have focused on the exploration of strategies to suppress pathogen QS, to remove biofilms formed by pathogenic bacteria and to inhibit the secretion of pathogenic toxic factors. On plant roots, where discontinuous domains govern microbial colonization, the spatial patterns of cell dispersion can vary and can therefore alter the QS distance^20^. Similarly, bacteria can also generate cell aggregates in the 3D environment of the human body. These cell aggregates can feature different spatial distributions, which also influence bacterial QS. Stacy *et al*. demonstrated that the virulence of a pathogenic bacterium was enhanced by modulating its spatial location at the infection site^1^. However, experimental studies of the mechanism by which the spatial distribution of cells influences bacterial QS have been hindered by a lack of an effective model. In the present study, we developed an experimental model based on hydrogel microcapsules with a 3D structure to allow similar numbers of cells to form different spatial distributions in a finite space and examined the influence of the spatial distribution of cells on bacterial QS.

Cell aggregate structure was beneficial for bacterial QS compared to cells without aggregates. When the same concentration of exogenous signaling molecules were added, owing to the similar mass transfer property of both aggregates and no aggregates microcapsules, the different bioluminescent response is likely due to the spatial distribution of cell aggregates. It suggested that the structure of cell aggregates in a finite space may also influence cellular response through the restriction of autoinducers, which may lead to increased self-communication. The reason for higher levels of autoinducers is likely due to the shorter cell-cell communication distance, reduced diffusivity and positive feedback leading to higher production rates. Positive feedback can make the response steeper and the activation threshold lower, facilitating self-activation^21^. The higher expression level of *luxN* and *luxQ* genes in aggregates group can also support this speculation (Fig. 2). Microorganisms, as the simplest cells, cannot distinguish specific information from the complex environment, only perceiving changes in their surroundings via molecular transmission signals^5^. However, they also have their own defense system. Microbial cells can resist stress factors through biofilm formation^22^,^23^, which is inseparable from the cell aggregate structure^24^,^25^. It is assumed that cells tend to grow collectively and form cell aggregates to better withstand environmental changes compared to single cells. This tendency toward collective growth may be one of the reasons that cell aggregates display stronger QS capacity.

**Figure 8:**
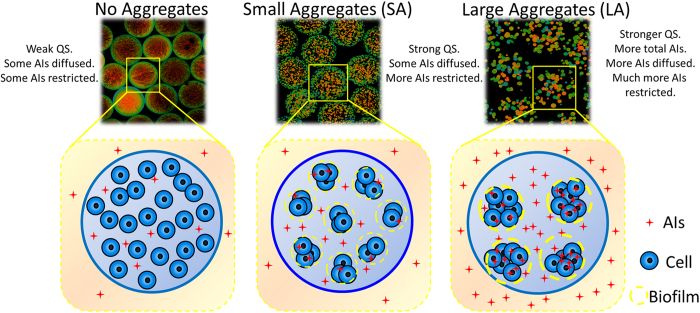
A schematic illustration of the mechanism of the influence of spatial distribution on bacterial QS. In the no-aggregate group, despite a sufficient number of cells, only relatively weak QS phenotype is triggered because the communication distance is relatively large. In the SA group, relatively strong QS phenotype is triggered by the same number of cells. As cell aggregates form, more AIs are restricted in the aggregates, and the cell-cell communication distance is decreased because of the aggregation. In the LA group, much stronger QS occurs. The increased number of cells in the aggregate unit decreases the cell-cell interaction distance and produces a higher AI concentration. The AIs, although diffused out of the microcapsules, can be restricted in the aggregates and can ultimately have a huge impact on QS. Thus, spatial distribution plays a crucial role in bacterial QS.

Assuming similar total numbers of cells, the LA group, which had fewer aggregates but more cells in each aggregate, had a stronger QS capacity than did the SA group, which had more aggregates but fewer cells in each aggregate. The LA group exhibited higher bioluminescence intensity and up-regulated QS related genes expression. The AI-2 induction of LA group was significantly higher than both no aggregates and SA group no matter inside or outside of the microcapsules. There may be two reasons behind the more total amount of AIs in LA group. One is that the larger aggregates may restrict a larger amount of AIs, which in turns increases the QS phenotype. It can be supported by the biofilm-like structure generating outside of the aggregates, which can hinder the diffusion of AIs, thus improving the local concentration of AIs inside the aggregates (Fig. 8). Another is that the more cells in each aggregate may improve the self-induction and facilitate AIs secretion. Finally, the strength of QS increased as the number of cells in the aggregates increased, consistent with the natural law of “strength in numbers”.

## Conclusion

In summary, bacterial QS in different cell spatial distributions in a finite space was investigated based on a 3D structure model. Cell aggregates exhibited much stronger QS capacity than did cells with a uniform distribution. In addition, cells that formed large aggregates (LAs) grew together to form a new social community, thus decreasing the cell-cell communication distance and increasing the local AI concentration. Consequently, microbial QS was facilitated and enhanced in LA group. These findings provide insight into the nature of QS and offer new tools to study bacterial QS, particularly in complex discontinuous environments and with respect to pathogenic biofilm inhibition or host-microbe interactions.

### Experimental procedure

#### Bacterial strains and materials

Wild type *Vibrio harveyi* BB120 (ATCC BAA-1116) and the mutant strains *V. harveyi* BB170 (*luxN*::tn5Kan, ATCC BAA-1117) and BB152 (*luxM*::tn5Kan, ATCC BAA-1119) were purchased from the American Type Culture Collection (ATCC). The culture medium was Autoinducer Bioassay medium^26^, and the culture temperature was 30 °C. Sodium alginate was purchased from the Chemical Reagent Corporation (Qingdao, China), and the viscosity was greater than 0.02 Pa s when it was dissolved to form a 1.5% (w/v) aqueous solution at 20 °C. ε-Poly-L-lysine (ε-PLL, Mw = 16,000) was kindly donated by Prof. Shiru-Jia (Tianjin University of Science and Technology). All other reagents and solvents were of analytical grade and were used without further purification.

#### Microencapsulation

Alginate/ε-poly-L-lysine microcapsules were prepared as a model to establish different spatial distributions of cells for the study of bacterial QS. The microcapsules were prepared as described previously with some modifications^27^,^28^. A specific number of *V. harveyi* cells was suspended in 1.5% (w/v) sodium alginate followed by the formation of cell-entrapped microbeads using a high-voltage electrostatic generator (YD-05, Dalian Institute of Chemical Physics, Chinese Academy of Science, China). Then, a 0.05% ε-poly-L-lysine solution was used to form a microcapsule with a semipermeable membrane^18^,^29^.

The no-aggregate group was obtained by directly encapsulating 1 × 10^10^ CFU/mL cells in the microcapsules. The aggregate group was prepared by encapsulating 1 × 10^8^ CFU/mL cells in the microcapsules, followed by culture for about 12–16 hours to obtain the same number of cells as in the no-aggregate group. Cells in both groups were inoculated from the same seed culture, which were both grown into their logarithmic phase. The only distinguish between the aggregates and no-aggregate group was the seed cells were either cultured outside or inside the microcapsules to reach the same cell density.

The large aggregate (LA) and small aggregate (SA) groups were prepared by encapsulating 5 × 10^6^ CFU/mL and 5 × 10^8^ CFU/mL cells in the microcapsules, respectively, and the two groups were cultured for 16 and 8 hours respectively to finally obtain 1 × 10^10^ CFU/mL cells with different cell aggregate structures (the growth curves of LA and SA groups were shown in Fig. 3c). All the cells encapsulated in the microcapsules were in their middle logarithmic phase.

#### Mass-transfer properties

Fluorescein isothiocyanate (FITC, 10 μg/mL) was chosen to assess the mass transfer properties of the microcapsules. The molecular weight of FITC is 389 Da, which is similar to the molecular weights of the AIs of *V. harveyi* (AI-1 187 Da and AI-2 193 Da). On the purpose of investigating the effect of capsule membrane on the AIs diffusion, the empty microcapsules were suspended in the FITC solution at a ratio of 1:10 (v/v) and immediately scanned under a confocal laser scanning microscope (CLSM, Leica TCS-SP2, Germany). Alternatively, the empty microcapsules were suspended in the FITC solution (0.1 mg/ml, 1:10 v/v) for 2 hours to obtain equilibrium between the FITC inside and outside the microcapsules; the microcapsules were then moved to a saline solution to scan the outside fluorescence intensity. All images were captured along the maximum cross-section of the microcapsule for comparison.

The mass transfer properties of the cell-entrapped microcapsules were determined using FITC diffusion experiment as well. The cell-entrapped microcapsules were suspended in the FITC solution (10 μg/mL) at a ratio of 1:10 (v/v) and immediately scanned under CLSM every 30 s for 15 to 30 min. The fluorescence intensity of FITC (λex = 488 nm; λem = 530 nm) inside the microcapsules was determined by selecting several random sections to analyze the average signal counts.

#### Morphological characterization

Optical images of the *Vibrio harveyi* cells in the microcapsules were obtained under a Nikon Eclipse TE2000 Inverted Research Microscope (Nikon, Japan). A confocal laser scanning microscope (CLSM, Leica TCS-SP2, Germany) equipped with a blue laser source (Ar 488 nm/5 mW) and an inverted microscope (Leica, DMIRE2, Germany) were employed to characterize the morphology of the cell aggregates in the microcapsules. The cells in the microcapsules were stained with a LIVE/DEAD Baclight^TM^ Bacterial Viability Kit (L7012, Invitrogen, USA). The sizes of the aggregates were determined with ImageJ software following release from the microcapsules. The images of released aggregates were transferred to gray level, and the size of aggregates was calculated by diameter measurement.

#### Bioluminescence assay

All bioluminescence assays were performed using a microplate reader (BioTek, Synergy H1, USA) in luminescence mode. A 0.1-mL aliquot of sample was specifically added to a 96-well plate to detect bioluminescence production. The data are reported as the relative bioluminescence intensity, i.e., the signal count (arbitrary units) normalized to the OD value. Each sample was measured in at least three independent wells, and each data point represents three independent samples. Samples from both groups were washed with saline solution to attenuate the inherent bioluminescent differences, and 100 μL of sterile supernatant from *V. harveyi* (an overnight culture) was then added as an exogenous AI for several hours to detect the AI response capacity.

#### AI-2 detection

AI-2 was detected using a biological method as described previously with some modifications^30^: As a first step, 0.5 mL of the culture supernatant from each group was centrifuged at 8000 × g at 4 °C for 5 min and filtered through a sterile 0.22-μm filter; the cell-free supernatants were stored at −20 °C for no more than 2 days before detection. For the subsequent assays, the reporter *V. harveyi* BB170 strain was cultured overnight at 30 °C with aeration and diluted at 1:5000 into fresh AB medium. Then, 180 μL of the diluted solution was added to each well, followed by the addition of 20 μL of cell-free supernatant from the experimental samples. The negative control was 20 μL of sterile AB medium, and the positive control was 20 μL of cell-free culture supernatant from BB152 that had been cultured overnight. The samples and controls were incubated in a 96-well plate in a rotary shaker at 170 rpm and 30 °C for approximately 6 hours. Every half hour, the bioluminescence intensity was measured using a microplate reader (BioTek, Synergy H1, USA) in luminescence mode. Each sample was measured in at least three independent wells, and each data point represents three independent samples. The AI-2 activity was quantified as the relative fold induction of AI-2, which was calculated as follows:


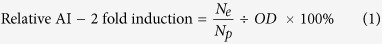


where Ne represents the bioluminescence of the experimental samples and Np represents the bioluminescence of the positive control.

In order to detect the AIs inside and outside of the microcapsules, 0.5 mL suspensions of cell-entrapped microcapsules in the culture medium were taken to detect the outside AIs. Then, all medium was removed and the same volume of saline solution was added to maintain the constant volume. Microcapsules were physically broken along in the saline water using homogenizer, and then the solution was centrifuged at 8000 × g at 4 °C for 5 min and filtered with a sterile 0.22-μm filter to detect the AIs inside the microcapsules.

#### Quantitative real-time PCR

Approximately 10^8^ cells were harvested from each group for RNA extraction. Total RNA was extracted using RNAiso Plus reagent (TaKaRa, Japan) according to the manufacturer’s protocol. The RNA quantity and quality were determined using a NanoPhotometer (Implen, Germany) by measuring the absorbance ratio at 260 nm/280 nm. The RNA was then treated with Recombinant DNase I (Takara, Japan) and the PrimeScript^TM^ RT Reagent Kit with gDNA Eraser (Takara, Japan) to degrade the genomic DNA and prevent DNA contamination. Reverse transcription was performed using PrimeScript^TM^ RT Master Mix (TaKaRa, Japan) according to the manufacturer’s instructions in a 5333 PCR Mastercycler (Eppendorf, Germany). The qRT-PCR amplifications were performed with at least 3 biological replicates using 2X SYBR^®^ Premix Ex Taq^TM^ II (DRR081A, TaKaRa, Japan) and the CFX96 Real-Time System (Bio-Rad, USA). The housekeeping gene RNA polymerase A subunit (*rpoA*) was used as a control for normalization. The primer sequences are shown in Tab. S1. The transcriptional levels of the target genes were normalized to that of the *rpoA* gene to obtain relative expression levels.

#### Biofilm formation assay

Aggregates with different sizes but same cell density in 0.1 mL microcapsules were gently released by chemical method as previously described^31^. Briefly, 0.1 mL microcapsules were added into 1.0 mL ethylene diamine tetraacetic acid (EDTA, 0.1 M, pH 10.0) to dissolve the hydrogel structure and release the cell aggregates. Then, the gently collected aggregates were stained with 0.1% (w/v) crystal violet for 5 min at room temperature. Unattached stain was removed by low speed centrifugation, and the aggregates were washed three times with saline solution. The bacteria-bound crystal violet was dissolved in 1.0 mL 95% ethanol, and the absorbance was determined at 595 nm using UV spectrophotometer^32^. All assays were performed in triplicate and repeated three times.

#### Statistical analysis

All experiments were performed at least in triplicate, and the results are expressed as the mean ± standard deviation (SD). Significant differences between groups were analyzed by t-test. A p value < 0.05 was considered statistically significant, and the statistical analysis was performed by one-way ANOVA test in Originpro85 software.

All the gene sequence information used in this manuscript was from NCBI and the primer sequences were designed by TaKaRa Company, which were shown in the Supplementary materials.

## Additional Information

**How to cite this article**: Gao, M. *et al*. A crucial role for spatial distribution in bacterial quorum sensing. *Sci. Rep.* **6**, 34695; doi: 10.1038/srep34695 (2016).

## Supplementary Material

Supplementary Information
