# Supplementary material for: A crucial role for spatial distribution in bacterial quorum sensing
Source: Sci Rep. 2016 Oct 4;6:34695. doi: 10.1038/srep34695 (PMC5048177; doi:10.1038/srep34695)
Supplement: Supplementary Information [file srep34695-s1.pdf]

Supplementary Information

A crucial role for spatial distribution in bacterial quorum sensing

Meng Gao,[1,3] *Huizhen Zheng,* [1,3] *Ying Ren,* [1,3] *Ruyun Lou,* [1,3] *Fan Wu,* [1,3] *Weiting Yu,* [1,]* *Xiudong Liu,*[2,]* *Xiaojun Ma*[1]

[1] Dalian Institute of Chemical Physics, Chinese Academy of Sciences, Dalian 116023, P.R. China

[2] College of Environment and Chemical Engineering, Dalian University, Dalian Economic Technological Development Zone, Dalian 116622, P.R. China

[3] University of the Chinese Academy of Sciences, Beijing 100049, P.R. China

AUTHOR INFORMATION

*Corresponding Authors:

Dr. Weiting Yu

Laboratory of Biomedical Material Engineering

Dalian Institute of Chemical Physics (DICP)

Chinese Academy of Sciences (CAS)

457 Zhongshan Road

Dalian 116023, P.R. China

Tel: 0086-411-84379139

Fax: 0086-411-84379096

E-mail: yuwt@dicp.ac.cn

Table S1. Primer sequence of *Vibrio harveyi*.

Gene	Sequence (5’-3’)
luxR	CCACGTTGTGCGTCAGTTCT F
	GCGCTCCACTCAAACCAAAC R
luxS	CGAAGCAAATCGCGAAGAAC F
	TAGTCGATGCGTAGCTCTCT R
rpoA	CGTAGCTGAAGGCAAAGATGA F
	AGCTGGAACATAACCACGA R
luxN	TATCGGCCTCGTTAGCTTTG F
	GATACCGGCGATCATGTAGTT R
luxQ	GCTCCTTTCTGATACCACCTAC F
	CCTCAAGTTCTGGCGAATCT R
luxM	GATGCGATCACGTTGAGTAATCTT F
	AGACAAAGACAATAGCGACAGCA R

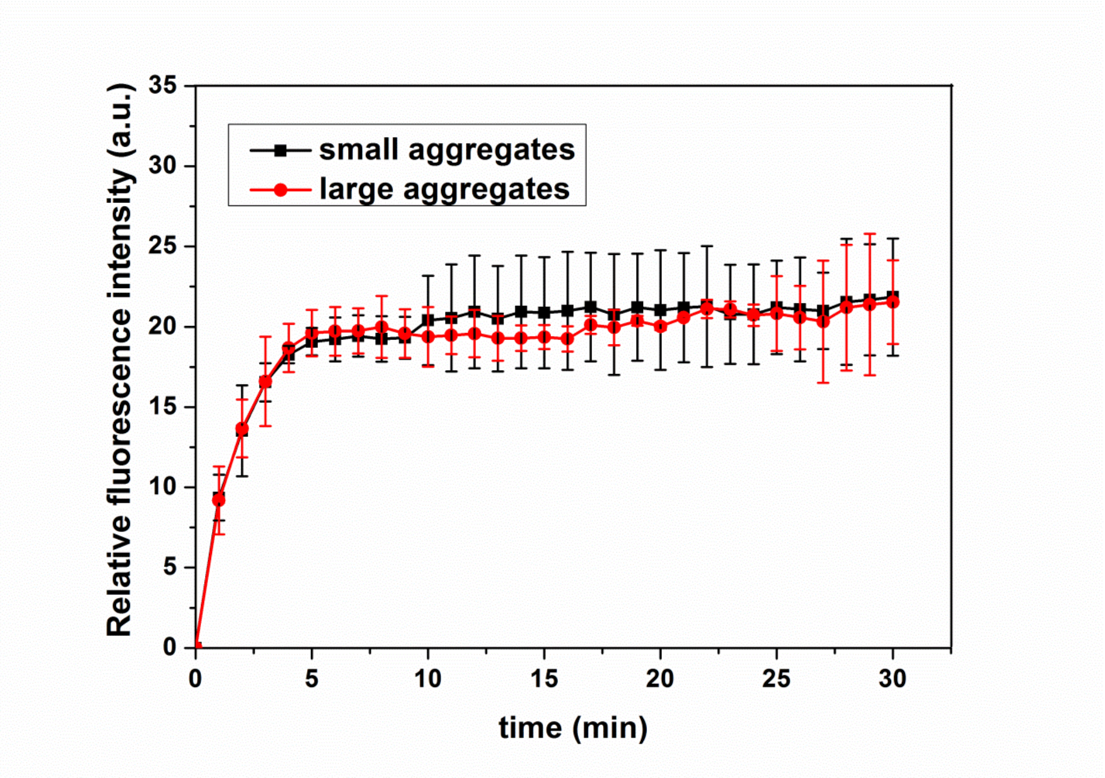

Figure S1. The mass transfer properties of microcapsules with LA and SA groups. FITC, which is as small as autoinducers, was chosen as the model molecule and can freely diffuse into these two kinds of microcapsules without significant difference.
